# Identification of a Three-Glycolysis-Related lncRNA Signature Correlated With Prognosis and Metastasis in Clear Cell Renal Cell Carcinoma

**DOI:** 10.3389/fmed.2021.777507

**Published:** 2022-01-10

**Authors:** Tinghao Li, Hang Tong, Junlong Zhu, Zijia Qin, Siwen Yin, Yan Sun, Xudong Liu, Weiyang He

**Affiliations:** ^1^Department of Urology, The First Affiliated Hospital of Chongqing Medical University, Chongqing, China; ^2^Department of Urology, Bishan Hospital of Traditional Chinese Medicine, Chongqing, China

**Keywords:** glycolysis, prognosis, clear cell renal cell carcinoma, lncRNA, LINC00342

## Abstract

The clear cell renal cell carcinoma (ccRCC) is not only a malignant disease but also an energy metabolic disease, we aimed to identify a novel prognostic model based on glycolysis-related long non-coding RNA (lncRNAs) and explore its mechanisms. With the use of Pearson correlation analysis between the glycolysis-related differentially expressed genes and lncRNAs from The Cancer Genome Atlas (TCGA) dataset, we identified three glycolysis-related lncRNAs and successfully constructed a prognostic model based on their expression. The diagnostic efficacy and the clinically predictive capacity of the signature were evaluated by univariate and multivariate Cox analyses, Kaplan–Meier survival analysis, and principal component analysis (PCA). The glycolysis-related lncRNA signature was constructed based on the expressions of AC009084.1, AC156455.1, and LINC00342. Patients were grouped into high- or low-risk groups according to risk score demonstrated significant differences in overall survival (OS) period, which were validated by patients with ccRCC from the International Cancer Genome Consortium (ICGC) database. Univariate Cox analyses, multivariate Cox analyses, and constructed nomogram-confirmed risk score based on our signature were independent prognosis predictors. The CIBERSORT algorithms demonstrated significant correlations between three-glycolysis-related lncRNAs and the tumor microenvironment (TME) components. Functional enrichment analysis demonstrated potential pathways and processes correlated with the risk model. Clinical samples validated expression levels of three-glycolysis-related lncRNAs, and LINC00342 demonstrated the most significant aberrant expression. *in vitro*, the general overexpression of LINC00342 was detected in ccRCC cells. After silencing LINC00342, the aberrant glycolytic levels and migration abilities in 786-O cells were decreased significantly, which might be explained by suppressed Wnt/β-catenin signaling pathway and reversed Epithelial mesenchymal transformation (EMT) process. Collectively, our research identified a novel three-glycolysis-related lncRNA signature as a promising model for generating accurate prognoses for patients with ccRCC, and silencing lncRNA LINC00342 from the signature could partly inhibit the glycolysis level and migration of ccRCC cells.

## Introduction

Renal cell carcinoma (RCC) ranks seventh of the most serious types of malignancies worldwide (1), maintaining the highest morbidity among kidney cancer and demonstrating an upward trend over the past 30 years (2). Clear cell renal cell carcinoma (ccRCC) is the most common pathologically RCC subtype that accounts for 85–90% of cases (3). Although ccRCC is less malignant and more sensitive to adjuvant therapy than non-ccRCCs, there are nearly 30% of patients with ccRCC who showed recurrence or metastasis after surgery (4). The unpleasant outcomes of ccRCC may be attributed to an insufficient understanding of histological and molecular heterogeneities, which are related to carcinogenesis and metastasis. Hence, new biomarkers are urgently needed to predict the prognosis of patients with ccRCC and to better understand the biological mechanisms.

Tumor cells always transform their metabolic pathways to sustain their unrestricted growth and progression under rough tumor microenvironments (TMEs). As one of the main energy source substances, glucose metabolism switching from oxidative phosphorylation to aerobic glycolysis is one hallmark of cancer cells, also known as the Warburg effect (5). Glycolysis may be a potential target for ccRCC diagnosis and treatment, the aberrantly glycolytic level is positively correlated with the progression of cancer cells (6, 7), prompting that glycolysis could be reliable in predicting the clinical outcome of patients with ccRCC.

Long non-coding RNA (lncRNA) is a kind of non-coding RNAs that contain longer than 200 nucleotides. Recently, lncRNA has been reported as key regulators of various cellular processes in multiple diseases, such as the development and progression of cancers (8, 9). Therefore, lncRNA can be reasonable as prognostic biomarkers in ccRCC and emerging evidence supports this point (10).

Here, we identified a three-glycolysis-related lncRNA signature from The Cancer Genome Atlas (TCGA) database to predict the prognosis of patients with ccRCC. The predictive performance of our signature was investigated in several ways and validated by testing cohort from International Cancer Genome Consortium (ICGC) database. A nomogram based on the three lncRNA signatures and clinical features was constructed. The connection between the three glycolysis-related lncRNAs and tumor-infiltrating immune cells (TIICs) in the TME was assessed, functional enrichment analyses were employed to explore underlying biological processes and mechanisms implicated in ccRCC. For the first time, we explored the overexpression of lncRNA LINC00342 in ccRCC cells and cancer tissues. Its crucial role in ccRCC glycolysis and metastasis was further investigated.

## Materials and Methods

### Data Source

The transcriptome expression profiles and clinical information were obtained from TCGA (https://portal.gdc.cancer.gov) as for the training group and ICGC (https://dcc.icgc.org/) as for the testing group. The details of the included cohorts' clinical characteristics, such as overall survival (OS), survival time, age, sex, grade, stage, tumor size, distant metastasis, and lymph node metastasis, are listed in Supplementary File S1. Patients with incomplete data or vague living status were excluded from our study. Glycolysis-related gene expression profiles named “Hallmark glycolysis” and “Kyoto Encyclopedia of Genes and Genomes [KEGG] glycolysis gluconeogenesis” were downloaded from Molecular Signatures Database (http://www.gsea-msigdb.org) and also listed in Supplementary File S2.

### Differentially Expressed Gene Analysis

Genes were identified as protein-coding genes or lncRNAs according to their Ensembl IDs (Supplementary File S3). DEGs between the normal and tumor samples from the TCGA database were identified *via* the differential expression analysis using the R package “LIMMA” (log2 foldchange [logFC] >2 and *p* < 0.01).

### Construction of Three-Glycolysis-Related lncRNA Signature and Prognostic Model

With the use of VENN-4 (http://bioinformatics.psb.ugent.be/webtools/Venn/), we got the intersection of the DEGs and 245 glycolysis-related genes, which contained 35 glycolysis-related DEGs (Supplementary File S4). Pearson correlation analyses were employed to identify glycolysis-related lncRNAs. The correlation was calculated according to the expression value between lncRNAs and glycolysis-related DEGs (cut-off criteria: |R| > 0.5 and *p* < 0.05). After Cox regression analysis realized by survival package of R, we finally got three prognosis-associated glycolysis-related lncRNAs. Hazard ratio (HR) < 1 represents good OS outcomes, and HR >1 represents poor OS outcomes (Table 1). We utilized these three glycolysis-related lncRNAs to construct our prognostic model, the formula that calculates the risk score of patients with ccRCC is constructed as follows: $\left(\text{RiskScore}\right)\;=\;\overset{n}{\underset{i=1}{\sum }}\left(Expi*\;Coei\right)$ (11). In this formula, “n” means the number of prognostic genes, “Expi” means the expression of lncRNA i, and “Coei” means the regression coefficient of the corresponding lncRNA obtained by the multivariate Cox regression model.

**Table 1:** Glycolysis-related lncRNAs associated with the prognosis of patients with ccRCC.

**Gene symbol**	**Aliases**	**Ensemble ID**	**Location**	***p***	**HR**
AC009084.1	HSALNG0112137	ENSG00000265408	chr16:66,976,615-66,997,159	3.71446453710324E−06	0.662221433943955
AC156455.1	MLXIP-1	ENSG00000256546	chr12:122,063,289-122,078,514	0.000603152141481603	1.36774809449761
LINC00342	NCRNA00342	ENSG00000232931	chr2:95,807,051-95,835,568	1.03872677603238E−06	1.6831127994666

*ccRCC, clear cell renal cell carcinoma*.

### Evaluation of the Three Hypoxia-Related lncRNAs Prognostic Signature

With the use of the aforementioned formula, patients with ccRCC from the training or testing cohort were divided into high-risk or low-risk subgroups by the median risk score. The OS of each cohort between the high- and low-risk groups was compared by the survival curve. The diagnostic efficacy and clinicopathological characteristic of the 3-glycolysis-related lncRNAs signature were evaluated by the receiver-operating characteristic (ROC) curves. In addition, the efficiency of the risk score to independently predict the survival of the training cohort was assessed by univariate and multivariate Cox regression analyses.

### Principal Component Analysis and Gene Set Enrichment Analysis

Principal component analysis was carried out with a “scatterplot3D” R package to demonstrate the expression of ccRCC samples in different subgroups. GSEA was used to detect the enrichment of different functional phenotypes between the low-risk group and the high-risk group. Normalized enrichment score (NES) >1.6 and *p* < 0.05 were considered statistically significant.

### Estimation of Clinical Independence and Construction of the Nomogram

The “rms” R package was utilized to consolidate the clinical features with the risk scores, to construct a nomogram for clinical prognostic prediction of patients with ccRCC. According to the different variable feature, a horizontal straight line was drawn to ascertain the points for each variable, and the total points of each patient were calculated by adding the points of all variables together, which were normalized to a distribution from 0 to 100. Then we built 3- or 5-year calibration plots and time-dependent ROC curves to evaluate the performance of our nomogram.

### Functional Enrichment Analysis

The “LIMMA” R package was utilized to analyze the potential pathways and process of DEGs in training and testing cohorts, respectively. Gene ontology (GO) and KEGG enrichment analyses for DEGs were conducted using the “clusterProfiler” package in R. GSEA software (version 4.0.1) was performed to identify differences in the set of genes enriched in high-risk groups. The canonical pathway gene sets (c2.cp.v7.4.symbols.gmt) were downloaded from the Molecular Signatures Database (http://www.gsea-msigdb.org/gsea/msigdb/collections.jsp). Gene set permutations were performed 1,000 times for each analysis.

### Immune Cells Infiltration Analysis

The R software's CIBERSORT algorithm was utilized to determine the profile of TIICs (including 22 immune cells) in all tumor samples of the TCGA cohort. The relationship of TIICs and risk score was analyzed by the LIMMA package. Single-sample gene set enrichment analysis (ssGSEA) was conducted to measure the different capacity levels of defending tumor infiltration between the high- and low-risk groups.

### Construction of the Co-expression Network

The lncRNA-miRNA-mRNA co-expression network was constructed by Cytoscape, to research the correlation between the hypoxia-related lncRNAs and their target microRNAs and the downstream mRNAs. LncRNA-miRNA connections (Supplementary File S5) were predicted by data downloaded from miRcode. TargetScan, miRDB, and mieTarBase databases were chosen to screen out microRNAs that target mRNAs at the same time, selected mRNAs were also taken intersection with hypoxia-related mRNAs to get reliable hypoxia-related lncRNA-targeted mRNAs.

### Cell Culture and Treatment

Four strains of ccRCC cell lines (including 786-O, RCC-JF, RCC-23, and Caki-1) and human renal tubular epithelial cell HK-2 were obtained from American Type Culture Collection (Manassas, VA, USA). The 786-O, RCC-JF, and HK-2 were incubated in RPMI 1640 medium (Corning Inc., Corning, NW, USA), RCC-23 was incubated in Dulbecco's modified Eagle's medium (DMEM) (Gibco, Amarillo, TX, USA), and Caki-1 was cultured with McCoy's 5A medium (Biological Industries, Israel). All media were supplemented with 10% fetal bovine serum (Gibco, Thermo Fisher Scientific, MA, USA), and cells were incubated at 37°C in 5% CO_2_. All small interfering RNAs (siRNAs) targeting human lncRNA LINC00342 (si-LINC00342-1, si-LINC00342-2) or scrambled negative control (si-NC) were designed and synthesized by GenePharma (Shanghai, China; Table 2), transfected into the cell lines with Lipofectamine 2000 (Invitrogen, Carlsbad, CA, USA), according to the instructions of the manufacturer.

**Table 2:** Sequences of small interfering RNA or primer sequences for quantitative real-time PCR.

**Designation**	**Genes**	**Sequences **(5^′^−3^′^)****	**Organism**
Primer	Lnc-AC156455.1	F: -TGCCCAGGAGCTAGAAATGT-	
		R: -CTGGGGGTTAAGGGTCAAGT-	
	Lnc-AC009084.1	F: -TTCTTTGGGGGCAACTACATT-	
		R: - GGCCTCGCACACGAC-	Homo-sapiens
	Lnc-LINC00342	F: -TGTGGAGGCTAAAAGCGGG-	
		R: -AGAAAGTCCTGCCATGCACAA-	
	β-actin	F: -AGAAAATCTGGCACCACACCT-	
		R: -GATAGACAGCCTGGATAGCA-	
si-RNA	si-Lnc-LINC00342-1	F: -GAAGAUGCUAACUAGAAUAACTT-	
	si-Lnc-LINC00342-2	R: -GUUAUUCUAGUUAGCAUCUUCTT-	Homo-sapiens
		F: -GGAUGAAUUGACAGAAGUAGGTT-	
		R: -CCUACUUCUGUCAAUUCAUCCTT-	

### Quantitative Real-Time PCR

Total RNAs were drawn out from different groups of 786-O cells or tissues processed by different treatments by using TRIzol (Invitrogen, CA, USA). PrimeScript RT reagent kit (TaKaRa, Osaka, Japan) was used to reverse-transcribe RNA (1 μg) to cDNA. Real-time quantitative PCR (qPCR) was performed with SYBR Green (TaKaRa, Tokyo, Japan) on an ABI 7500 Real-Time PCR System (Applied Biosystems, Waltham, MA, USA), the entire process lasted for 45 cycles following the instructions of the manufacturer. Each sample was repeated three times in detection. Data were standardized to β-actin using the 2^−ΔΔCt^ method. The primer sequences used for the real-time qPCR are listed in Table 2.

### Immunoblots

Total protein was drawn out using radioimmunoprecipitation assay lysis buffer (Beyotime, China) containing 1 mmol/L phenylmethylsulfonyl fluoride (PMSF) and 1 mmol/L β-Glycerophosphate sodium salt hydrate, which were both purchased from Selleck (Houston, TX, USA). The concentration of protein samples was detected by a BCA protein quantitative kit (Beyotime, China). 10% sodium dodecyl sulfate-polyacrylamide gel was chosen for total protein separation and transferred to nitrocellulose membranes (Millipore, Burlington, MA, USA). Membranes were blocked by skimmed milk for 2 h at room temperature and then incubated with primary antibodies, such as anti-E-Cadherin (1:1,000, #3195; Cell Signaling Technology [CST], Beverly, MA, USA), anti-N-Cadherin (1:1,000, #13116; CST), anti-β-actin (1:5,000, 20536-1-AP; Proteintech, Rosemont, IL, USA), anti-c-Myc (1:1,000, A5011; Bimake, Beijing, China), anti-β-catenin (1:1,000, #8480; CST), anti-p-β-catenin (1:1,000, #4176; CST), anti-Vimentin (1:1,000, #5741; CST), and anti-Slug (1:1,000, #9585; CST). These antibodies were added and incubated overnight at 4°C. Then enhanced chemiluminescence reagents (Millipore, USA) were used to assess protein expression, which was normalized to the corresponding bands for β-actin.

### Lactate Production and Glucose Consumption Measurement

Lactate and glucose concentrations in the culture supernatants were detected using the Lactate Assay kit (Solarbio, Beijing, China) and the Glucose Assay kit (Solarbio), respectively, according to instructions of the manufacturer, and absorbance values were measured at the corresponding absorbance. The results were normalized by the number of cells in each sample in the culture plates, lactate production and glucose consumption were calculated by comparison with the normal medium.

### Cell Migration Assays and Wound Healing Assays

The longitudinal migrate ability was assessed by trans-well assay. Cells transfected with different sequences were inoculated with a density of 1 × 10^4^ cells per well in the upper chamber processed by disparate treatments, resuspended by 500 μl media without containing FBS, and seeded into the insert chamber. The chambers were incubated in a 24-well plate at 37°C with 5% CO_2_ for 12 h. Migrated cells that attached to the substratum of the membrane were observed and photographed after fixed stained. The number of migrating cells represented migration activities in different groups. The lateral migrate ability was assessed by wound healing assay. At 48 h post-transfection, cells were grown to confluence in 6-well plates. Wounds were scratched in each well by sterile pipette tips. The wells were washed by PBS and cultured in RPMI-1640 without serum to inhibit cells proliferation. After 0 and 24 h of incubation, the migration status was assessed by photographing the same location in the wells. Then, the area of the healed wound represented migration activities in different groups.

### Clinical Data for Human Tissue Specimens and Bioinformatic Analysis

Eleven pairs of cancer tissues and adjacent normal tissues were collected from patients who underwent radical nephrectomy and post-operative pathological diagnosis was confirmed for ccRCC, at the First Affiliated Hospital of Chongqing Medical University.

### Statistical Analysis

The R software (version 3.5.1) with corresponding packages, SPSS 20.0 statistical software, and GraphPad Prism 7 were used for statistical analyses. Unless specified otherwise above, *p* < 0.05 denoted statistically significant differences. The PERL programming language (version, 5.30.2, http://www.perl.org) was used to process data. The Kaplan–Meier method and the log-rank test were performed to compare the OS between the high- and low-risk groups. By employing “GSVA,” the ssGSEA-normalized ccRCC DEGs were compared to a genome (R-package). Experiments were independently repeated three times and representative images are performed in figures. Results of analyses are performed as the mean ± SD. Student's *t*-tests were performed to compare the differences between two groups, and significance levels were set to p^*^ < 0.05, p^**^ < 0.01, p^***^ < 0.001, or non-significant (ns).

## Results

### Identification of Glycolysis-Related lncRNAs in ccRCC

**Figure 1:**
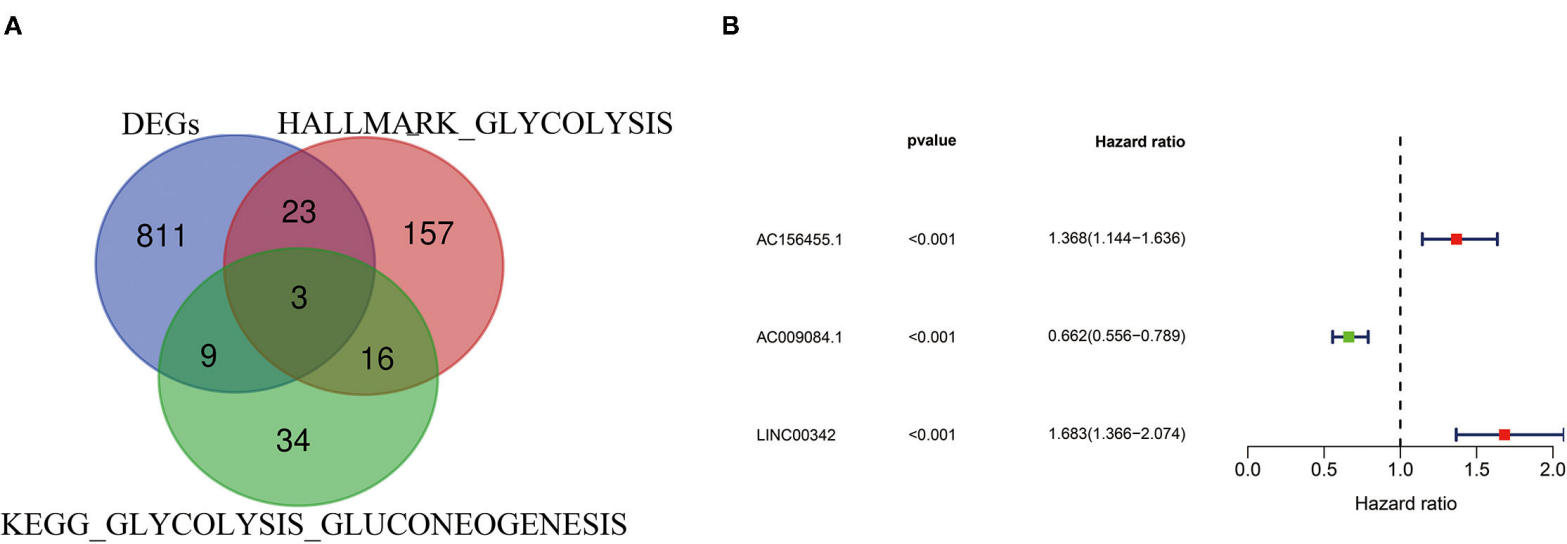
Construction of glycolysis-related lncRNA signature in ccRCC. **(A)** Two glycolysis-related gene sets (Hallmark glycolysis and KEGG glycolysis gluconeogenesis) and mRNA expression of DEGs were taken at the intersection. **(B)** 3-glycolysis-related lncRNAs were extracted by multivariate Cox regression analysis. ccRCC, clear cell renal cell carcinoma; KEGG, KEGG, Kyoto Encyclopedia of Genes and Genomes; DEG, differentially expressed genes.

Differentially expressed genes in ccRCC specimens (*n* = 539) and normal renal specimen (*n* = 72) were extracted from TCGA database (cut-off criteria: *p* < 0.01 and |logFC| > 2), i.e., 846 different expression mRNAs and 169 lncRNAs (Supplementary Figure S1A; Supplementary File S3). By analyzing the intersection between different expression mRNAs and two glycolysis-related gene sets, we finally got 35 glycolysis-related different expression mRNAs (Figure 1A; Supplementary File S4). With the use of Pearson correlation analysis between the different expression lncRNAs and the glycolysis-related different expression mRNAs, we initially got 38 glycolysis-related different expression lncRNAs. After performing univariate Cox analysis, we identified 15 candidate lncRNAs (*p* < 0.05) closely related to OS. Next, 3-glycolysis-related lncRNAs were identified as independent prognostic factors after multivariate Cox regression analysis between the 15 lncRNAs and the survival data (Figure 1B; Table 1).

### Verification of the Glycolysis-Related lncRNAs Prognostic Model

**Figure 2:**
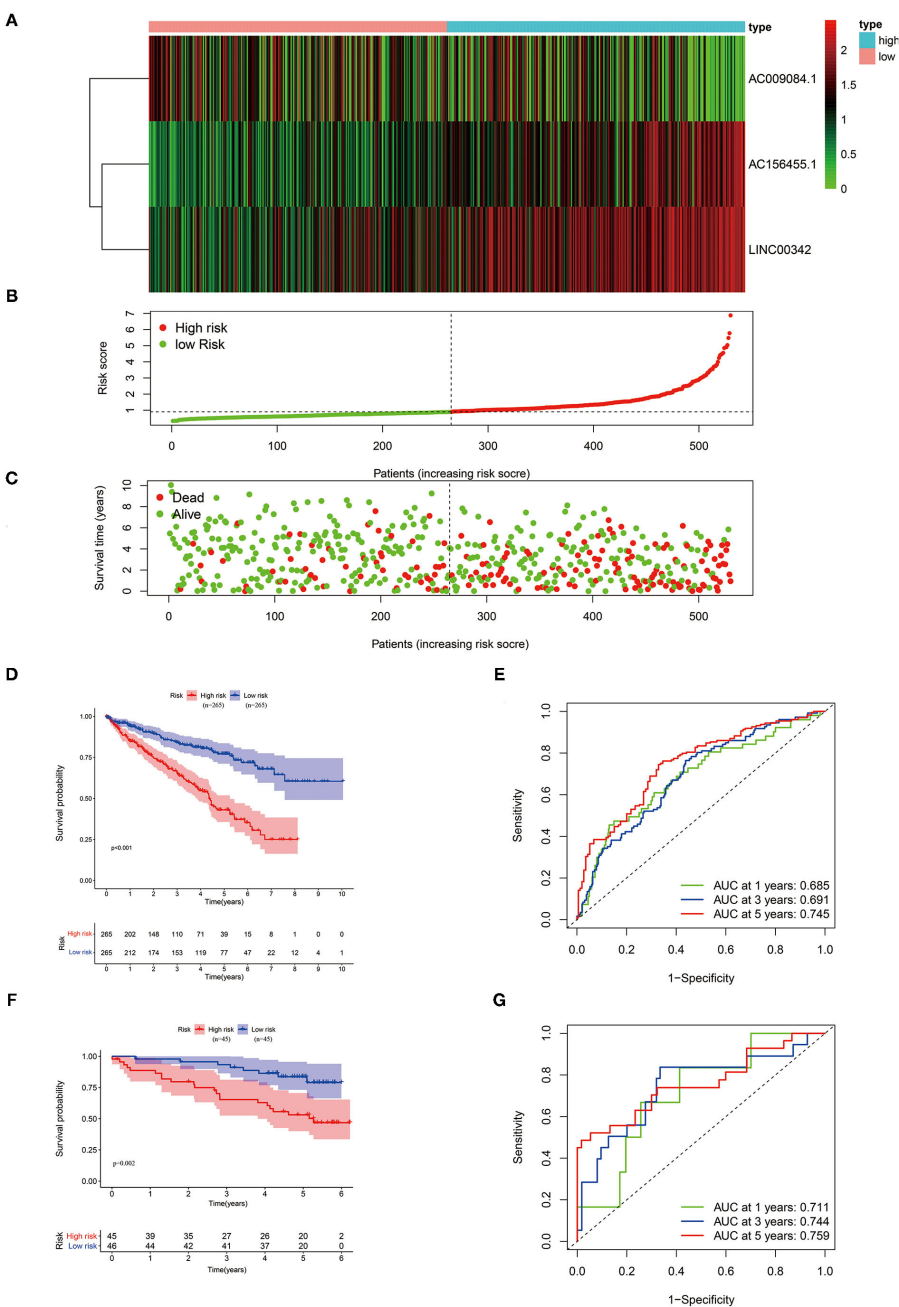
Validation of our three-glycolysis-related lncRNA signature in ccRCC. **(A)** The expression of three-glycolysis-related signature in 530 patients with ccRCC of the training cohort were demonstrated in the heatmap. **(B)** Patients were divided into high- or low-risk groups sorting by risk scores. **(C)** The survival status and survival time of patients with ccRCC listed by risk scores. **(D,F)** The survival curve analysis of three-glycolysis-related lncRNAs signature, the OS period in the low-risk group was longer than the high-risk group in both training and validation cohort. **(E,G)** Time-dependent ROC curves analysis exhibited the diagnostic efficacy of our three-glycolysis-related lncRNA signature in ccRCC of both training and validation cohort, for predicting the 1-, 3-, and 5-years OS. ccRCC, clear cell renal cell carcinoma; OS, overall survival.

With the use of multivariate Cox regression analysis, we constructed a glycolysis-related lncRNA signature for evaluating the prognosis of patients with ccRCC. The formula was as follows: Risk score = (AC156455.1 × 0.313165660795125) + (AC009084.1 × −0.412155286612758) + (LINC00342 × 0.52064493581871) (Table 1). Among the included lncRNAs, AC156455.1 and LINC00342 were prognostic risk factors, and AC009084.1 was a prognostic protective factor (Figure 2A). Then patients with ccRCC were sub-grouped into high-risk and low-risk groups according to our signature (Figure 2B). With increasing risk scores, the number of surviving patients with ccRCC was decreased (Figure 2C), and the high-risk group demonstrated a shorter OS period (Figure 2D). ROC curves performed that our 3-glycolysis-related lncRNAs were with high accuracy of diagnostic efficacy, in which the area under the ROC (AUC) was 0.745 for the 5-year prediction (Figure 2E). The prognostic value of our prognostic model was verified in our testing group. Patients with ccRCC sourcing from the ICGC database were divided into the high-risk groups (*n* = 45) and the low-risk groups (*n* = 45) according to our risk model. Kaplan–Meier analysis showed that survival probability in the high-risk group was lower than that in the low-risk group (*p* = 0.002; Figure 2F), ROC curves demonstrated that the AUCs for the testing group in 1-, 3-, and 5-year were 0.711, 0.744, and 0.759, respectively (Figure 2G). These results indicated that our glycolysis-related lncRNA signature was a valuable prognostic indicator in patients with ccRCC.

### The Glycolysis-Related lncRNA Signature Was Associated With Clinical Features and Progression of Patients With ccRCC

**Figure 3:**
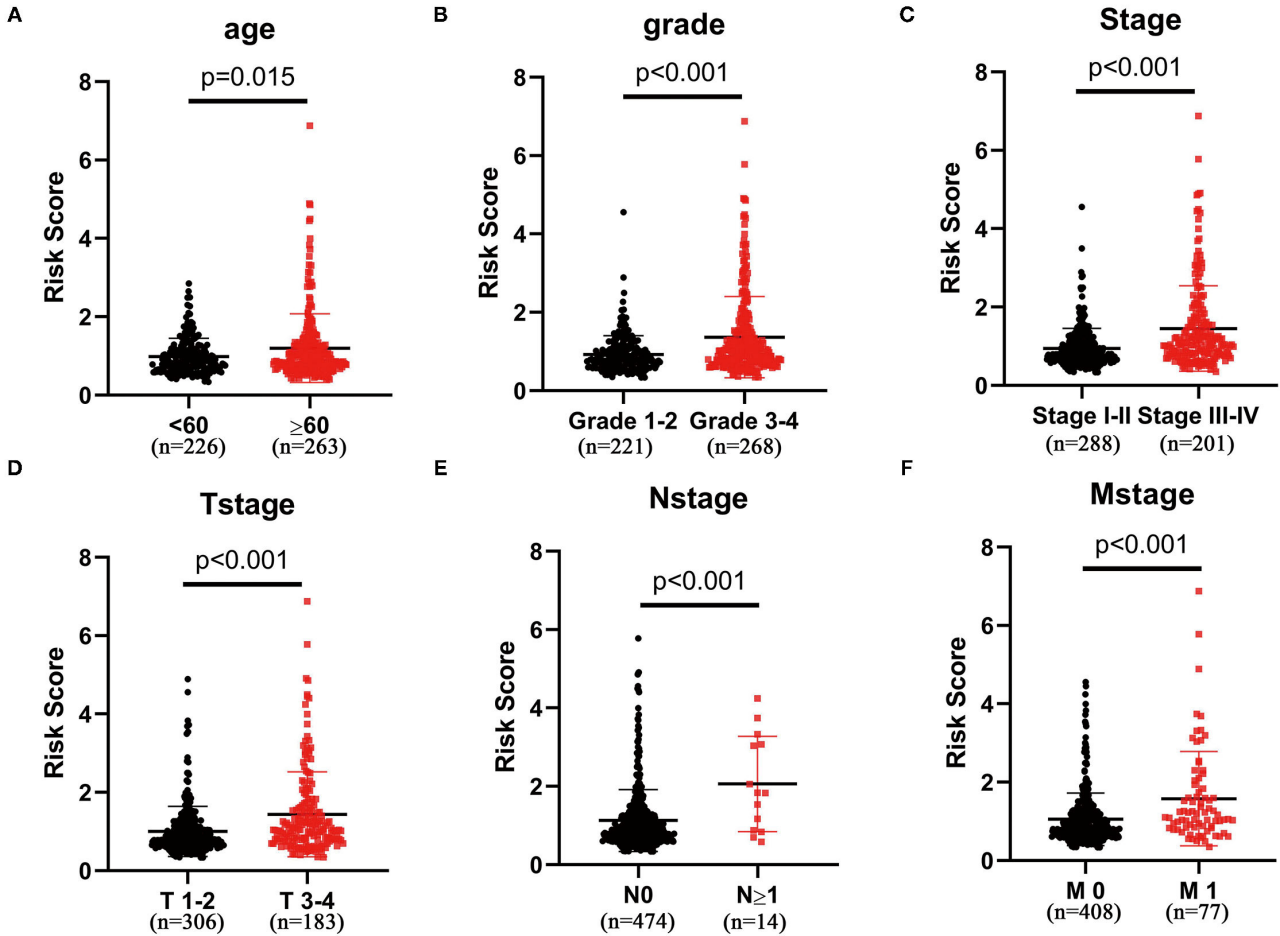
The three-glycolysis-related lncRNA signature was in accordance with clinicopathological parameters. The risk scores of our three-glycolysis-related lncRNA signature were closely related to clinicopathological parameters, such as **(A)** age (<60 vs. ≥60, *p* = 0.015), **(B)** grade (grade 1–2 vs. grade 3–4, *p* < 0.001), **(C)** AJCC stage (stage I–II vs. stage III–IV, *p* < 0.001), **(D)** T stage (T stage 1–2 vs. T stage 3–4, *p* < 0.001), **(E)** N stage (N stage 0 vs. N stage ≥1, *p* < 0.001), and **(F)** M stage (M stage 0 vs. M stage 1, *p* < 0.001).

After excluding patients without exactly clinical data, we analyzed the association between clinical features and glycolysis-related lncRNA signature in 489 patients with ccRCC. As shown in Figure 3, patients aged ≥65 years, Grade 3–4, Stage III–IV, T stage 3–4, N stage ≥1, and M stage 1 subgroups show significantly higher risk scores compared with patients aged <65 years (*p* = 0.015; Figure 3A), Grade 3–4 (*p* < 0.001; Figure 3B), Stage I–II (*p* < 0.001; Figure 3C), T stage 1–2 (*p* < 0.001; Figure 3D), N stage 0 (Figure 3E), and M stage 0 (*p* < 0.001; Figure 3F) groups, respectively. But there was no significant correlation between the risk scores and gender (*p* < 0.001; Supplementary Figure S1B).

### Cox Analysis Indicated the Glycolysis-Related lncRNA Signature as an Independent Factor

**Figure 4:**
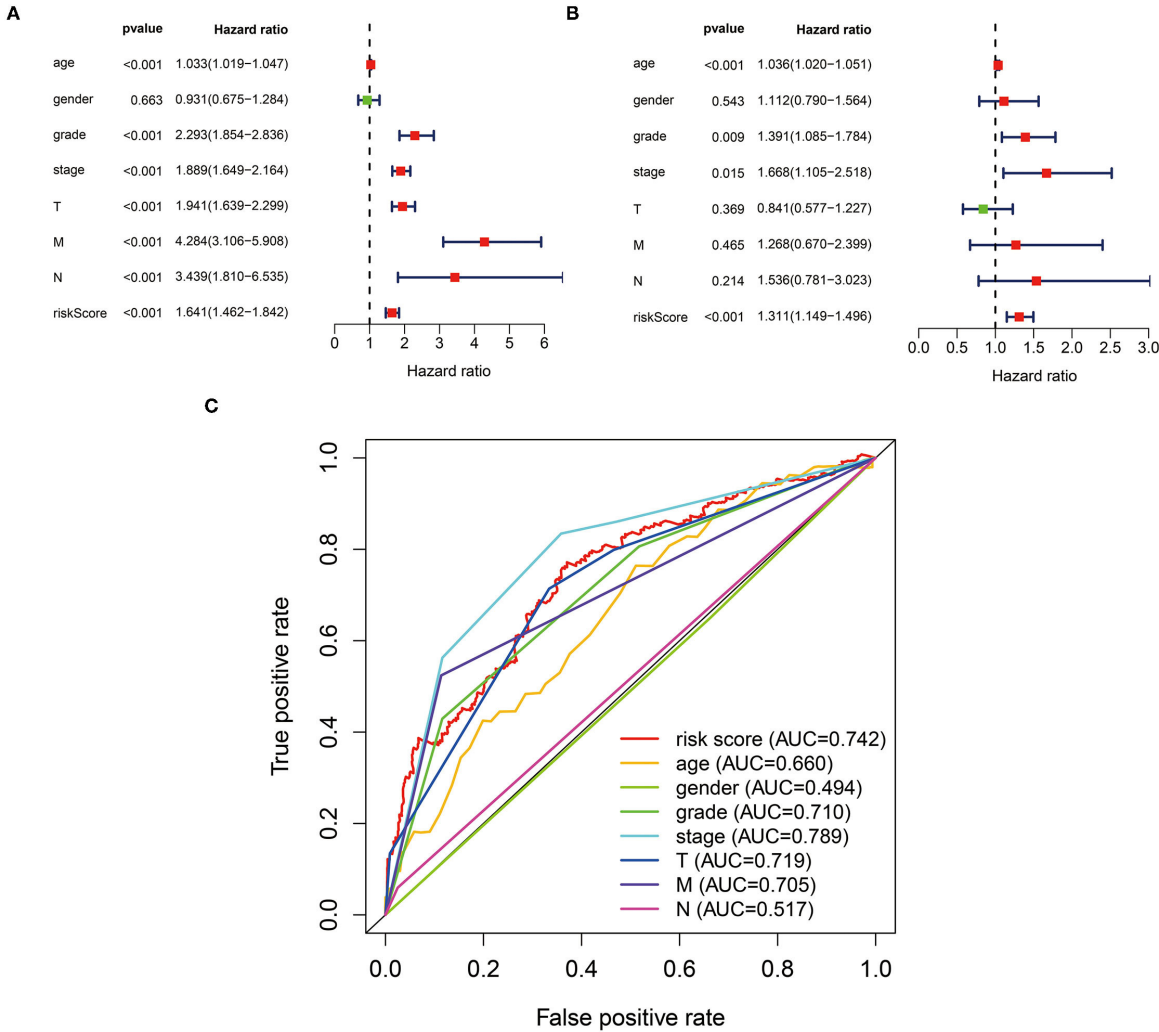
The three-glycolysis-related lncRNA signature could be an independent factor in ccRCC. **(A)** Univariate Cox regression analyses were used to explore the correlation between OS and clinicopathological parameters, and risk score. **(B)** Multivariate Cox regression analysis demonstrated that the age, grade, stage, and risk score were independent prognostic indicators for ccRCC patient's OS. **(C)** ROC curve analysis showed the prognostic accuracy of clinicopathological parameters and the glycolysis-related lncRNA prognostic risk score. ccRCC, clear cell renal cell carcinoma; OS. Overall survival.

In the univariate analysis, we found that age (HR = 1.033, 95% CI 1.019–1.047, *p* < 0.001), tumor grade (HR = 2.293, 95% CI 1.854–2.836, *p* < 0.001), AJCC stage (HR = 1.889, 95% CI 1.649–2.164, *p* < 0.001), T stage (HR = 1.941, 95% CI 1.639–2.299, *p* < 0.001), M stage (HR = 4.284, 95% CI 3.106–5.908, *p* < 0.001), N stage (HR = 3.439, 95% CI 1.810–6.535, *p* < 0.001), and risk score (HR = 1.641, 95% CI 1.462–1.842, *p* < 0.001) were all significantly associated with prognosis (Figure 4A). Multivariate analysis confirmed age (HR = 1.036, 95% CI 1.020–1.051, *p* < 0.001), tumor grade (HR = 1.391, 95% CI 1.085–1.784, *p* = 0.009), AJCC stage (HR = 1.668, 95% CI 1.105–2.518, *p* = 0.015), and risk score (HR = 1.311, 95% CI 1.149–1.496, *p* < 0.001) as independent prognostic factors (Figure 4B). The ROC curves also performed that the AUC value of the risk score was 0.742, which ranked the second highest (Figure 4C).

### Stratification Analyses of the Glycolysis-Related lncRNA Signature

**Figure 5:**
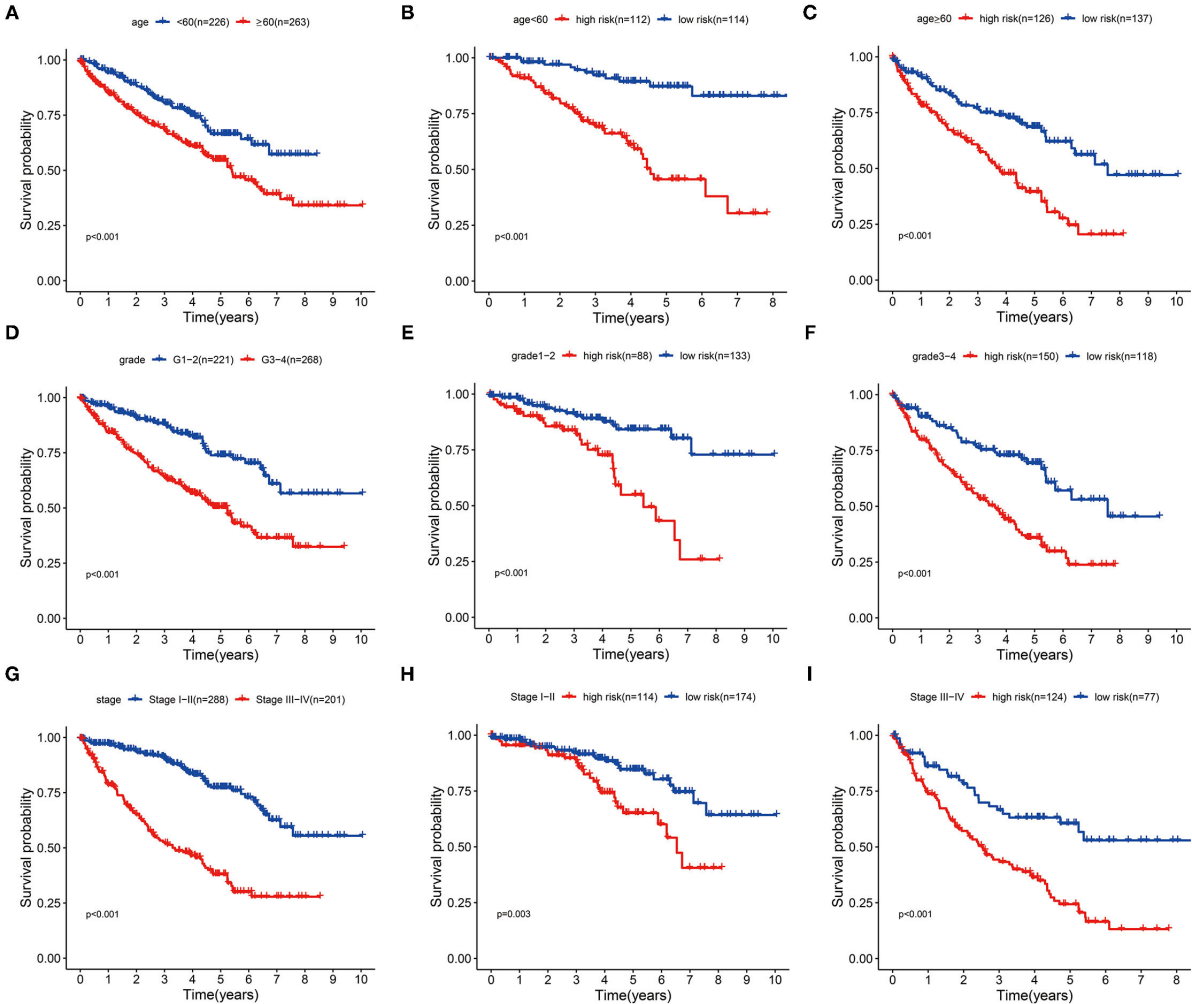
Stratification analyses of various clinicopathological parameters by Kaplan–Meier curves for the patients with ccRCC. **(A,D,G)** Survival curve analysis showed the OS rates of different groups stratified by age, grade, and stage. **(B,C)** Kaplan–Meier curves of OS in <60 or ≥60 patients stratified by high- and low-risk groups. **(E,F)** Kaplan–Meier curves of OS in grade 1–2 or grade 3–4 patients stratified by high- and low-risk groups. **(H,I)** Kaplan–Meier curves of OS in stage I–II or stage III–IV patients stratified by high- and low-risk groups. ccRCC, clear cell renal cell carcinoma; OS, overall survival.

Next, we extracted information about survival time and risk scores from 489 patients with ccRCC. In accordance with Multivariate analysis results, Kaplan–Meier survival curves demonstrated the OS time was longer in younger or lower malignancy patients (*p* < 0.001) (Figures 5A,D,G). Then we stratified these patients into different subgroups sorted by our three-glycolysis-related lncRNA signatures. The Kaplan–Meier survival analysis showed that ccRCC patients with high-risk score had shorter OS time compared with low-risk group, no matter age <60 (*p* < 0.001) or ≥60 (*p* < 0.001) (Figures 5B,C), grade 1–2 (*p* < 0.001) or grade 3–4 (*p* < 0.001; Figures 5E,F), stage I–II (*p* = 0.003), or stage III–IV (*p* < 0.001; Figures 5H,I). These findings indicated that our three-glycolysis-related lncRNA signatures could accurately predict the prognosis of patients with ccRCC in different gradations of age, grade, and stage.

### Principal Component Analysis

We utilized the PCA to explore the different distribution patterns between the low-risk group and the high-risk group in patients with ccRCC. In our three-glycolysis-related lncRNA signature gene sets (Supplementary Figure S1C), the low- and high-risk groups were obviously separated into two parts. While we did not detect the significant separation of the risk score based on the whole glycolysis-related lncRNAs gene set (Supplementary Figure S1D), glycolysis-related mRNA gene set (Supplementary Figure S1E), and all gene sets (Supplementary Figure S1F). It indicated that our three-glycolysis-related lncRNA signatures could distinct patients with ccRCC correctly compared with other distribution patterns.

### Construction and Validation of the Nomogram

**Figure 6:**
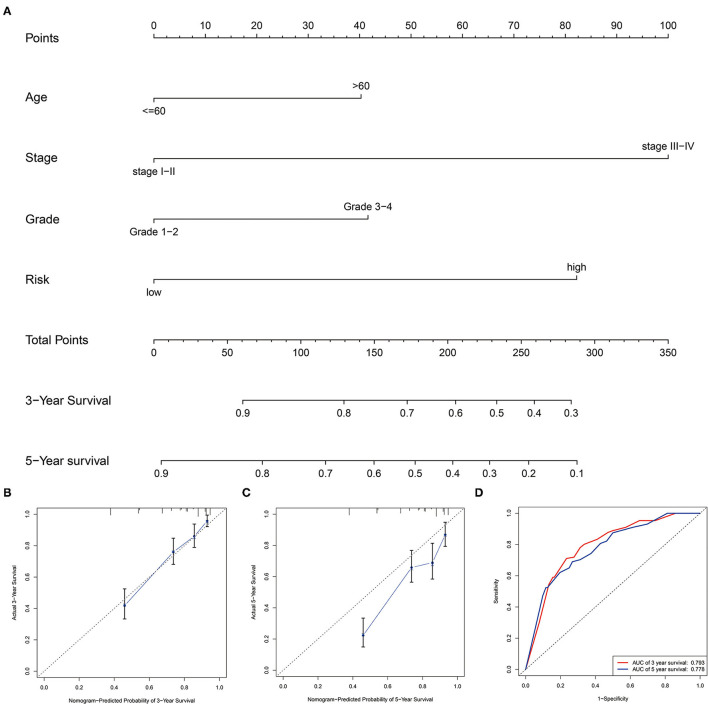
Construction and validation of the prognostic nomogram. **(A)** Construction of the nomogram was based on traditional clinical variables and the risk score. **(B,C)** Calibration plot for the internal validation of the nomogram at 3- and 5 years. **(D)** Time-dependent ROC curves showed AUC of the nomogram.

We generated a nomogram based on several independent predictive factors, according to our Multivariate analysis results, such as age, grade, stage, and our risk score, to predict the probability of 3- and 5-year OS rates of patients with ccRCC. Each factor is scored based on the proportion of contribution to survival risk as shown in Figure 6A. The calibration curve for the 3- and 5-year OS probability results indicated that the predicted survival rate is closely related to the actual survival rate (Figures 6B,C). The AUCs of the nomogram in the ROC curves were 0.793 and 0.778 at 3- and 5 years, respectively (Figure 6D).

### Functional Enrichment Analysis

**Figure 7:**
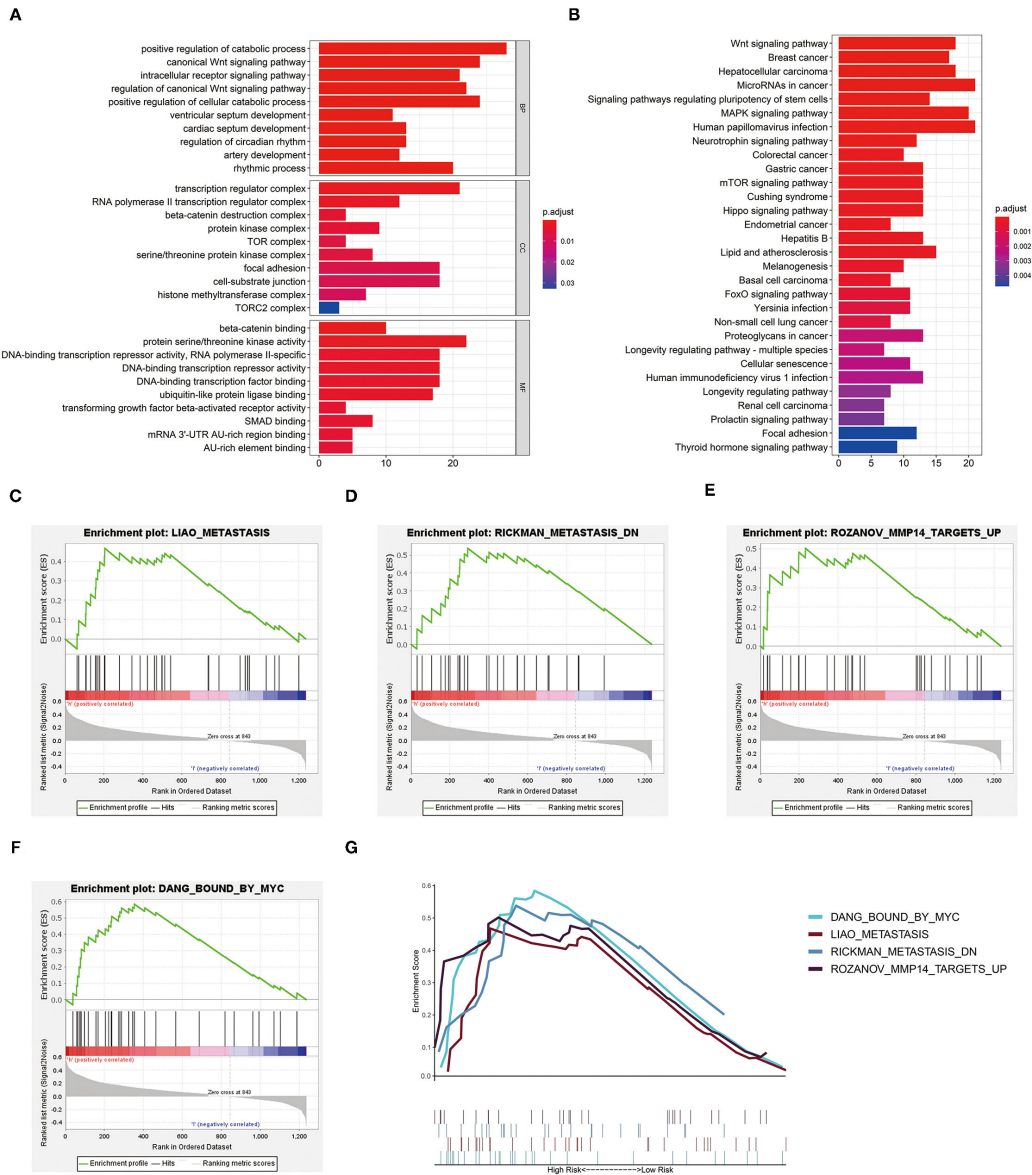
Functional enrichment analysis. **(A)** The GO term demonstrated the enriched molecular biological processes (BP), cellular components (CC), and molecular functions (MF). **(B)** The KEGG pathway analysis showed the significantly enriched pathways. **(C–G)** Canonical pathways gene sets enriched in the high-risk group, such as metastasis (NES = 1.66, *p* = 0.028), metastasis DN (NES = 1.62, *p* = 0.032), MMP4 targets up (NES = 1.67, *p* = 0.02), and bound by MYC (NES = 1.83, *p* = 0.01). KEGG, Kyoto Encyclopedia of Genes and Genomes; GO, gene oncology.

To explore the biological characteristics of the DEGs in high- and low-risk groups of our training cohort, GO enrichment and KEGG pathway analyses were utilized with the use of ClusterProfile R package. GO analysis indicated that DEGs were obviously enriched in some catabolic-related processes and regulators of the Wnt signaling pathway (Figure 7A). KEGG functional enrichment analysis suggested that DEGs were mostly enriched in the Wnt signaling pathway, focal adhesion, and RCC (Figure 7B). We also employed GSEA to conduct key GO term and pathway enrichment analysis of high-risk score ccRCC samples based on our three-glycolysis-related lncRNA signatures. Genes associated with high-risk score were mainly enriched in metastasis (NES = 1.66, *p* = 0.028), metastasis DN (NES = 1.62, *p* = 0.032), MMP4 targets up (NES = 1.67, *p* = 0.02), and bound by MYC (NES = 1.83, *p* = 0.01; Figures 7C–G).

### Involvement of Three-Glycolysis-Related lncRNA Signature in the Immune Cell Infiltrations

**Figure 8:**
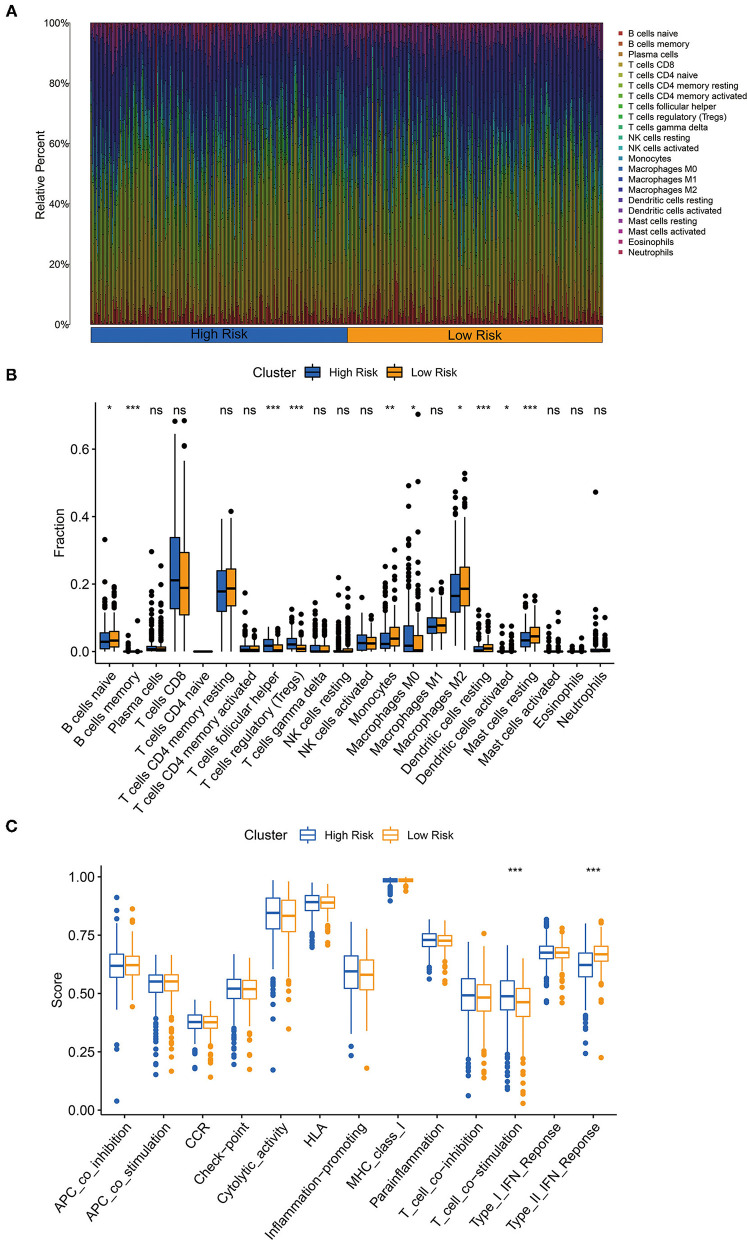
The three-glycolysis-related lncRNA signature was correlated with tumor-infiltrating immune cells (TIICs). **(A)** The CIBERSORT algorithm was utilized to calculate the relative percentage of 22 kinds of TIICs infiltrating in the high- or low-risk group, and were demonstrated in the bar plot. **(B)** Higher infiltrated levels of TIICs, such as memory B cells, follicular helper T cell, regulatory T cells (Tregs), and M0 macrophages, were observed in the high-risk group compared with the low-risk group (student's t-test, *p*ns > 0.05, p* < 0.05, p** < 0.01, p*** < 0.001). **(C)** Immune-related activities in high- and low-risk groups based on ssGSEA.

The CIBERSORT algorithm was chosen to explore the correlation of our three-glycolysis-related lncRNAs with TIICs in ccRCC. The proportion of each ccRCC patient's TIICs was analyzed using the CIBERSORT algorithm (Figure 8A). A comparison of the TIICs levels between the high- and low-risk groups demonstrated an elevated level of memory B cells, follicular helper T cell, regulatory T cells, M0 macrophages and decreased naive B cells, monocytes, resting dendritic cells, M0 macrophages, activated dendritic cells, and resting mast cells in the high-risk group (Figure 8B). Immune signature T cell co-stimulation exhibited significant activation and type II interferons (IFN) response inactivated in a high-risk group, according to a correlation analysis based on ssGSEA of training cohort (Figure 8C).

### The Overexpression of lncRNA LINC00342 Correlated With Prognosis and the Aberrantly Glycolytic Level of ccRCC

**Figure 9:**
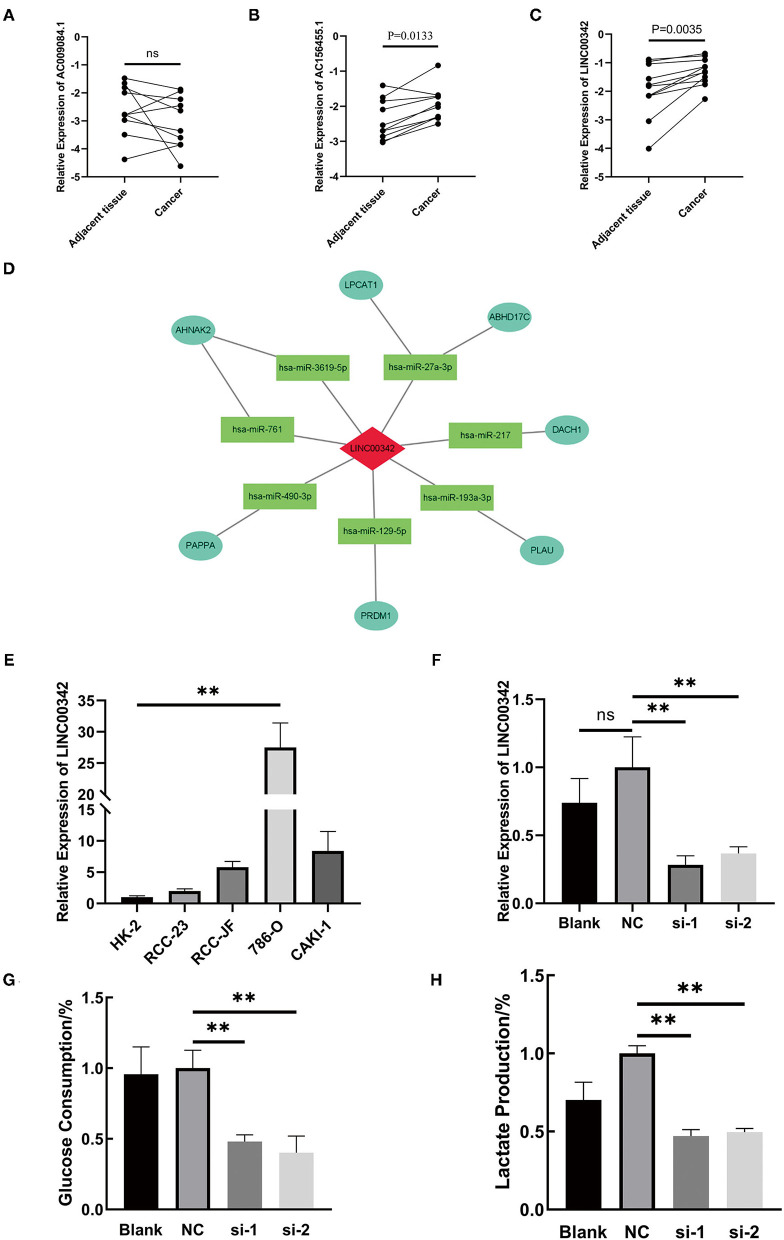
The crucial role of lncRNA LINC00342 played in the aberrantly glycolysis process of ccRCC. **(A–C)** Relative expression of three-glycolysis-related lncRNA in tumor and adjacent tissues from 11 clinical patients with ccRCC (paired student's t-test, *p*ns > 0.05). **(D)** Construction of LINC00342 related co-expression RNA regulation network based on differentially expressed miRNAs and DEGs. **(E)** The expression of LINC00342 was detected in HK-2 cells and other 4 kinds of ccRCC cells. LINC00342 expression levels were higher in all ccRCC cells than in HK-2, among which 786-O was the highest (student's t-test, *p*ns > 0.05, p** < 0.01). **(F)** After being transfected by siRNAs, we detected the knockdown efficiency of si-LINC00342s in 786-O. **(G,H)** Glucose consumption and lactate production ratio were detected in 786-O cells with different treatments. We found that after being silenced by two sequences of si-LINC00342s the glycolytic level in 786-O decreased a lot. ccRCC, clear cell renal cell carcinoma.

RNA was extracted from 11 ccRCC patients' cancer and adjacent tissues to explore the expression level of our three-glycolysis-related lncRNAs. As shown in Figures 9A–C, AC009084.1 is seemed to be expressed lower in cancer tissues but with no statistical significance. While AC156455.1 and LINC00342 demonstrated overexpression in cancer tissues, LINC00342 exhibited the most significant high expression. Survival analysis based on LINC00342 expression also showed its overexpression correlated with a shorter survival rate (Supplementary Figure S1G) from the TCGA database, indicating LINC00342 might be a crucial risk factor for ccRCC. To explore the potential mechanisms, we predicted LINC00342 connecting microRNAs by using miRcode. Results showed that 56 microRNAs could have interaction with LINC00342 (see Supplementary File S5). In addition, we predicted these miRNAs targeting proteins with the use of three databases (TargetScan, miRDB, and mieTarBase). Taking the intersection with DEGs we obtained before, we got lncRNA LINC00342 targeting 7 miRNAs, 7 mRNAs and constructed a co-expression RNA network (Figure 9D).

LINC00342 was generally overexpressed in renal carcinoma cell lines as compared with HK-2 cells, and the overexpression was most obvious in 786-O cells (Figure 9E). Then we silenced the expression of lncRNA LINC00342 in 786-O cells by two sequences of siRNAs (Figure 9F). After efficient inhibition on LINC00342 in contrast to wild type (WT) group or negative control (NC) group, the glucose consumption and lactate production rate in 786-O cells were decreased significantly (*p* < 0.01) (Figures 9G,H). These results indicated that LINC00342 overexpression is closely related to the aberrantly glycolytic level of ccRCC.

### Silencing lncRNA LINC00342 Inhibited 786-O Cells Migration Abilities

**Figure 10:**
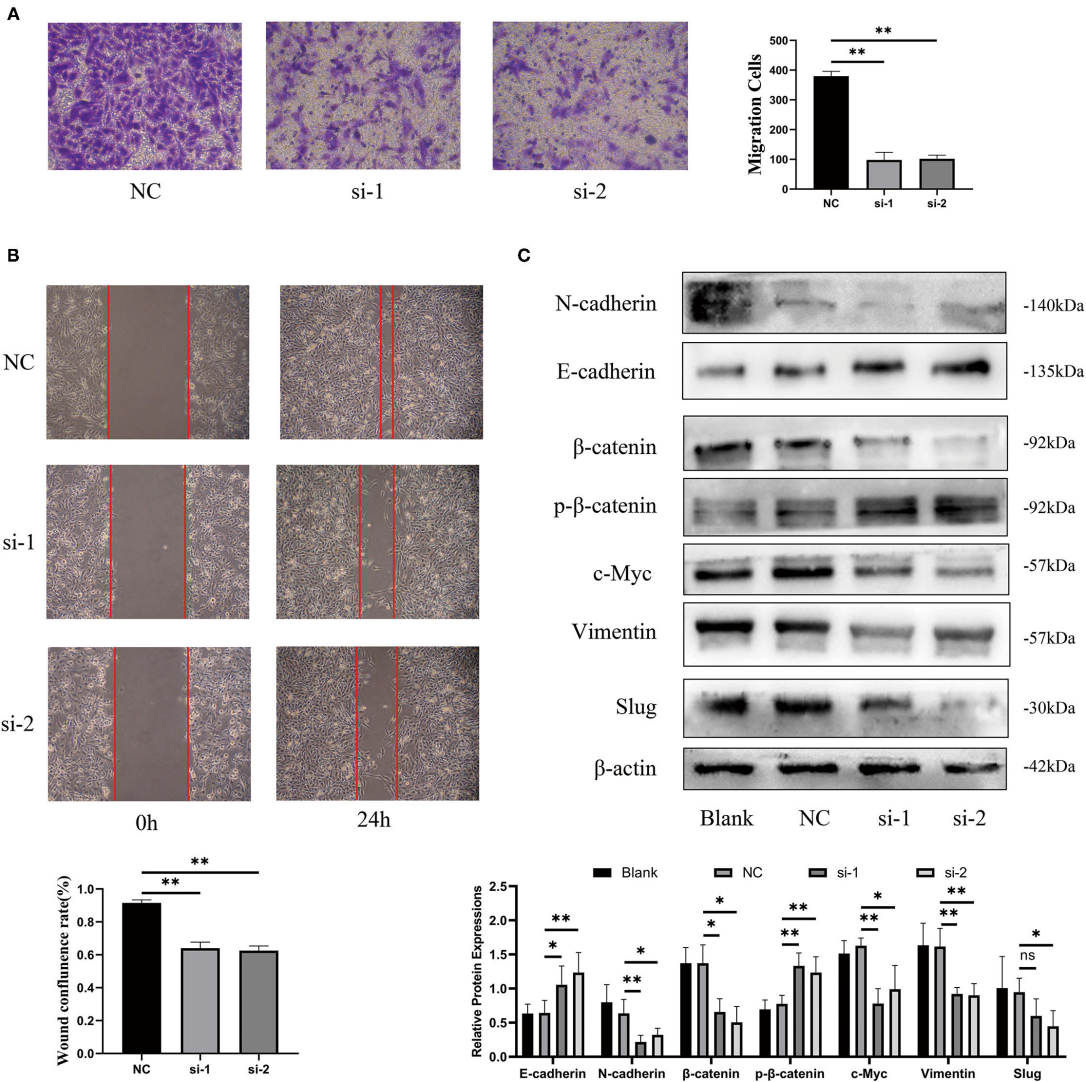
Silencing lncRNA LINC00342 inhibited migration abilities of 786-O cells. **(A)** Transwell assays were demonstrated that the longitudinal migration abilities of 786-O cells decreased significantly after silencing LINC00342 (student's t-test, *p*ns > 0.05, p* < 0.05, p** < 0.01). **(B)** Wound healing assay was certified that the lateral migration abilities of 786-O cells decreased a lot after silencing LINC00342. **(C)** Western blot assays of epithelial markers (E-cadherin), mesenchymal markers (N-cadherin, Vimentin, and Slug), and Wnt/β-catenin signaling pathway (β-catenin, phosphorylated β-catenin, and c-Myc) in 786-O cells transfected with si-LINC00342s or Vector. β-actin was taken as an internal control.

According to the aforementioned results, we speculated that the poor prognosis of high-risk patients with ccRCC, based on our three-glycolysis-related lncRNA signature, might be caused by a high risk of metastasis (Figures 7B–E). After silencing lncRNA LINC00342, we found that the longitudinal and lateral migration abilities in 786-O cells were diminished significantly in comparison with 786-O cells that transfected scrambled sequence as NC group, which were demonstrated by transwell assays and wound healing assays respectively (Figures 10A,B). It was reported that EMT is a critical step for ccRCC metastasis and relapse; consequently, western-blotting was employed to explore the effect of LINC00342 on EMT-associated proteins. Results were demonstrated that epithelial marker protein E-cadherin was increased after silencing LINC00342, while mesenchymal marker protein N-cadherin, Vimentin, and Slug were obviously decreased (Figure 10C). As DEGs mostly enriched in the Wnt signaling pathway and bound by MYC gene set were enriched in high-risk group (Figures 7A,B,F), we further detected effects of silencing LINC00342 on the Wingless (Wnt)/β-catenin pathway, which is reported as a crucial mechanism that promotes the occurrence of EMT. With the use of western-blot, we found β-catenin and c-MYC decreased a lot while phosphorylated β-catenin increased significantly in 786-O cells after silencing LINC00342 (Figure 10C). These results manifested that silencing the expression of lncRNA LINC00342 inhibited 786-O cells migration abilities. Mechanically, inhibiting LINC00342 overexpression could obstruct the EMT process in 786-O cells, partly by interfering Wnt/β-catenin pathway.

## Discussion

The ccRCC is not only a malignant disease among kidney cancer but also an energy metabolic disease, metabolic reprogramming has attracted extensive attention in the diagnosis and treatment of ccRCC. For cancer cells that survived in a TME of intermittently hypoxia and starvation, glucose metabolism transforms to glycolysis even in an aerobic environment is ubiquitous (5, 12). Metabolites produced by glycolysis support the excessive proliferation and metastasis of cancer cells, such as secreted lactate creates an acidic microenvironment which promoted cancer cells invasion abilities and absconding from chemotherapy (13, 14). Therefore, aberrantly elevated glycolysis in cancer cells was thought to be closely related to poor prognosis in malignancies, including ccRCC (6, 15). The mechanisms are still unclear, while there are emerging studies which tried to explore epigenetic modifications and transforms during the Warburg effect happens.

Though advances in surgery, radiotherapy, and chemotherapy, ccRCC as the most common pathologically subtype of RCC is still with a poor prognosis (16). The unpleasant outcomes may ascribe to an insufficient understanding of molecular heterogeneities. In addition to searching for effective treatment, it is vital important to make a reliable prediction. The AJCC staging system is the gold standard for evaluating ccRCC prognosis, but this criterion comes to be less comprehensive as understanding of the heterogeneity of tumor cells increased (15). In our present study, we focused on the abnormal glycolytic level in ccRCC and took an intersection between DEGs and glycolysis-related genes. With the use of selected 35 glycolysis-related different expression mRNAs and Pearson correlation analysis, we finally identified a novel prognostic model based on the expression of three-glycolysis-related lncRNAs, which were constructed by lncRNA AC156455.1, lncRNA AC009084.1, and lncRNA LINC00342. According to our three lncRNAs signature, we sorted patients with ccRCC into high- and low-risk groups. Kaplan–Meier analysis showed a shorter OS period in patients with high-risk scores both in the training and testing cohort. The three lncRNAs signature was confirmed by ROC curve analysis as a highly sensitive and specific prognostic marker in ccRCC. In addition, our signature was also associated with poor OS of patients with ccRCC in different subgroups especially age, tumor grade, and clinical stage. We also built a nomogram based on clinical features and glycolysis risk scores, which had better prognostic value and higher potential for clinical application than a single parameter. Therefore, our three-glycolysis-related lncRNA signature could be regarded as a complement to clinical patient prognosis prediction.

LncRNAs are untranslated RNAs but are crucial in several key molecular and biologic processes. There is increasing evidence indicating that lncRNA affects the occurrence and development of cancers mainly by regulating gene expression (17). LncRNA could regulate glycolysis metabolic reprogramming in several ways. LncRNAs may enhance the expression of glycolytic key enzymes to promote glycolytic level and progression in cancer cells. They interacted with transcription factor receptors, such as aryl hydrocarbon receptor, then promoted the transcriptional expression of glycolytic key enzymes, such as hexokinase 2 or pyruvate kinase M2, facilitated cancer cells progression (18, 19). LncRNA inhibited enzymes degradation by reducing the modification of key enzymes, such as ubiquitination (20). LncRNAs could also modulate glycolytic levels by interfering with micro-RNAs (miRNAs) and had further regulations on glycolysis-related pathways. Most lncRNAs acted as competing endogenous RNA or miRNA sponges to inhibit the functions of targeted miRNAs, then further affect downstream processes of glycolysis, and even form a positive feedback circuit between glycolytic reprogramming and metastasis in colorectal cancer (21). In our study, we constructed a ceRNA network to predict capable miRNAs and mRNAs related to LINC00342, which might be attributed to glycolytic reprogramming and the progression of ccRCC cells. LINC00342 was reported as an oncogene and correlated with cancer progression in various cancers. It regulated NPEPL1 expression by sponging miR-19a-3p and contributed to the growth and metastasis in colorectal cancer cells (22). LINC00342 could also promote the proliferation abilities of infantile hemangiomas by competitively binding miR-3619-5p with hepatoma-derived growth factors (23). However, the role of LINC00342 in ccRCC remained unknown. Our group found the overexpression of LINC00342 was in keeping with the poor prognosis of patients with ccRCC based on data from TCGA. *In vitro*, the expression of LINC00342 in ccRCC cell lines was generally higher than that in human renal tubular epithelial cells, in which 786-O expressed the highest. Silencing LINC00342 by siRNA inhibited the glycolytic level and migration abilities of 786-O cells, these results indicated the crucial role of LINC00342 in ccRCC glucose metabolic reprogramming and metastasis. EMT indicates the transformation from epithelial cells to mesenchymal cells, which attributes to enhancing the migration and motility of cancer cells (24). By detecting markers of epithelial cells and mesenchymal cells, the blocked EMT process could partially explain decreased migration abilities in 786-O cells after LINC00342 knockdown.

Aberrant glycolytic level of tumor cells could affect the interaction of cancer cells with the TME, leading to immune resistance or even escaping immune surveillance (25, 26). Recent studies have shown that lncRNAs play crucial roles in regulating immunity (27), which may be partly explained by the lncRNA interfering glycolysis process. Some lncRNAs brought by extracellular vesicles promoted cancer aerobic glycolysis and enhance their resistance to immune immunotherapy (28). LncRNA PVT1 could positively regulate glycolytic level elevation and CD4^+^ T cell activation (29). Hence, we speculated the correlations between the subtype of infiltrating immune cells and glycolysis-related lncRNAs. We found three-glycolysis-related lncRNA signature was significantly attributed to memory B cells, follicular helper T cell, regulatory T cells, M0 macrophages infiltration and suppressed naive B cells, monocytes, resting dendritic cells, M0 macrophages, activated dendritic cells, resting mast cells distribution in the high-risk group. The ssGSEA analysis pointed out immune signatures T cell co-stimulation activated and type II IFN response inactivated in a high-risk group. Results indicating our signature might involve in tumor immune microenvironment of ccRCC, works by arresting immune response and might be a contributor to ccRCC progression.

To explore potential mechanisms these three lncRNAs involved in glycolysis and poor progression, functional enrichment analysis were employed. In the present study, the GO term demonstrated DEGs mainly affected the catabolic-related processes and regulators of the Wnt signaling pathway. KEGG pathway enrichment analysis showed that DEGs were highly enriched in the Wnt signaling pathway, focal adhesion, and RCC. We also employed GSEA to conduct key oncology genes and pathways enrichment analysis of high-risk score ccRCC samples. As a result, the gene sets about metastasis and Wnt-related pathway regulators attracted our attention. Wnt/β-catenin signaling pathway was shown to be mainly involved in the Warburg effect and malignant progression of cancer cells (30, 31). It was reported that Wnt/β-catenin signaling induced an increase in glucose uptake and suppressed mitochondrial respiration, mechanically activating pyruvate carboxylase to support cancer cells proliferation (32). The Wnt pathway disruption led to inhibit mitochondrial oxidative phosphorylation of cancer cells, which was realized by down-regulating the expression and activity of pyruvate dehydrogenase kinase 1 (33). LncRNAs could suppress or elevate cancer cells glycolytic level through the Wnt/β-catenin signaling pathway, to sensitive cancer cells to chemotherapy or regulate their apoptosis (34, 35). Though there were few studies that reported Wnt/β-catenin signaling involved in glucose metabolic reprogramming in ccRCC, we found some connections between decreased glycolytic level and suppressed Wnt/β-catenin signaling pathway after silenced LINC00342.

However, we acknowledge that this research has significant limitations, these phenomenal were just observed by us and lack of sufficient interferes to make the conclusions more reliable. The low expression of lncRNA AC009084.1 in ccRCC tissue was insignificant in our real-world validation, which might be blamed for the insufficient sample size we collected. Herein, larger clinical trials are needed for further validation and longer observation, aiming to validate the different expressions of the three glycolysis-related lncRNAs and the prognostic value of our signature. Moreover, we only drew our attention to LINC00342 while ignoring the crucial role of AC009084.1 and AC156455.1, aimed to make an initial exploration but the current study is far from enough. Is there any relationship among these three lncRNAs or co-regulatory role, in regulating the aerobic glycolytic process and progression of ccRCC are still questions worth further investigation.

In conclusion, we identified a three-glycolysis-related lncRNA signature as a novel prognostic model. Our model exhibited superior predictive performance and could be utilized to independently predict the prognosis of patients with ccRCC or as a complement to clinical prognosis prediction for subgrouping patients with ccRCC. The relationships between the risk model and TIICs in ccRCC were also evaluated. By validation of clinical samples and ceRNA network constructing, we drew our attention to aberrant overexpression of lncRNA LINC00342 and the poor prognosis of ccRCC. For the first time, we found that after silencing LINC00342, glycolytic level and migration abilities in 786-O cells decreased a lot, which were explained by suppressed Wnt/β-catenin signaling pathway and reversed EMT process. Collectively, understanding the roles of the signature and the relationship between glycolysis and potential mechanisms can provide valuable insights for intensive research and the potential individualized treatment of patients with ccRCC.

## Data Availability Statement

The datasets presented in this study can be found in online repositories. The names of the repository/repositories and accession number(s) can be found in the article/Supplementary Material.

## Ethics Statement

The studies involving human participants were reviewed and approved by Ethics Committee of the First Affiliated Hospital of Chongqing Medical University. The patients/participants provided their written informed consent to participate in this study.

## Author Contributions

WH and TL conceived and designed the study. ZQ and SY collected the data. JZ and YS interpreted and analyzed the data. TL, XL, and HT wrote the manuscript. WH revised the manuscript critically. All authors contributed to the article and approved the submitted version.

## Conflict of Interest

The authors declare that the research was conducted in the absence of any commercial or financial relationships that could be construed as a potential conflict of interest.

## Publisher's Note

All claims expressed in this article are solely those of the authors and do not necessarily represent those of their affiliated organizations, or those of the publisher, the editors and the reviewers. Any product that may be evaluated in this article, or claim that may be made by its manufacturer, is not guaranteed or endorsed by the publisher.

## Supplementary Material

Supplementary Figure 1 **(A)** Heatmap was demonstrated the relative expression of DEGs in 539 ccRCC specimens and 72 normal renal specimens. **(B)** The relationship between risk scores of our three-glycolysis-related lncRNA signature and gender (men vs. women, *p* = 0.1216). **(C–F)** PCA between the high-risk group and low-risk group based on glycolysis-related lncRNA signature, glycolysis-related lncRNAs, glycolysis-related genes, and all gene sets, respectively. The red and green dots represent high-risk and low-risk genes, respectively. DEGs, differentially expressed genes. **(G)** Survival analysis based on LINC00342 expression on survival rate from TCGA database, *P* = 0.002.
